# Factors enabling community health workers and volunteers to overcome socio-cultural barriers to behaviour change: meta-synthesis using the concept of social capital

**DOI:** 10.1186/s12960-018-0331-7

**Published:** 2018-11-21

**Authors:** Nicole Mohajer, Debra Singh

**Affiliations:** 1 School of Public Health, College of Health and Medical Sciences, Haramaya University, P.O. BOX 138, 3020 Dire Dawa, Ethiopia; 2 Child and Adolescent Health Service, Health Department, Government of Western Australia, 189 Royal Street, East Perth, WA 6004 Australia

**Keywords:** Community health, Qualitative synthesis, Trust, Volunteer, Empowerment

## Abstract

**Background:**

Community-based health workers and volunteers are not just low-level health workforce; their effectiveness is also due to their unique relationship with the community and is often attributed to social capital, an area not well studied or acknowledged in the literature.

**Methods:**

A qualitative meta-synthesis was conducted using the SPIDER framework and based on critical interpretive synthesis. The protocol was registered with PROSPERO, ID = CRD42018084130. This article reports on the qualitative data extracted from the final 33 articles selected from 147 full-text articles on social capital and community-based health systems.

**Results:**

Three constructs were identified that enable community health workers to bring about changes in behaviour in the community: seeing their role as a service or a calling motivated by altruistic values, accompanying community members on their journey and the aim of the journey being empowerment rather than health. Community health workers feel under-resourced to provide for expectations from the community, to fulfil their non-health needs, to meet the expectations of their employers and to be able to deliver health services.

**Conclusion:**

The dichotomy of needs between the community and health services can be resolved if policy makers and programme designers examine the possibility of two cadres of community-based health workforce: full-time workers and part-time volunteers, with clear scopes of practice and supervision. Community health workers would primarily be concerned with task shifting roles demanded by programmes, and volunteers can focus on the wider empowerment-based needs of communities.

## Introduction

Community health workers (CHW) and volunteers (CHV) have been deployed globally as a local, low-cost health resource in communities with difficulty accessing mainstream health services. Their uptake has recently increased since the World Health Organization (WHO) started promoting ‘task-shifting’ or the inclusion of more medical skills and procedures to CHW workloads [1]. Community health *workers* are usually employed and paid a salary to perform specific tasks, whereas *volunteers*, who may perform the same tasks, receive incentives which can include small financial incentives. Community health volunteers usually do not have job security or assured pathways to future employment [2]. For example, in Ethiopia, the health extension workers are CHW, whereas the Women’s Development Army, who are usually from the poorest section of society [3], are CHV. As this distinction is not as clear in many countries, the notation CHW refers to trained, employed health workers and CH*W*/*V* is used to include both groups.

Upscaling CHW programmes has become a priority impelled by research estimates based on current evidence that more CHW will mean fewer deaths and less health expenditure burden [4, 5]. The selection, training and career paths of CHW in each country have been informal arrangements through non-government organisations (NGOs) or parallel training, separate from other health and medical professional courses, reinforcing the perception that they are a stop-gap solution in low resource settings until ‘real’ health professionals can take over [1, 6].

One of the reasons for the effectiveness of community-based health workers and volunteers (CH*W*/*V*) is thought to be due to social capital generated by their relationships of reciprocity and trust with the community as compared with the top-down bureaucracy of the health systems [7, 8]. Unlike the training of doctors, nurses and other ‘professional’ health workers, this relationship is an essential component of their effectiveness, as seen in a review of CHW and productivity [9] and also one of the major motivations for CHW to continue working despite poor pay, limited supervision, lack of supplies and few career opportunities [10]. Despite the number of papers about social capital and its documented effect on health, the exact nature of social capital and the causality of its health effects remain uncertain [11].

The purpose of this review was to synthesise and critique published literature for evidence of the underlying constructs that cause improvements in health in community systems using community health workers, based on the hypothesis that social capital plays a central role. It was felt to be an opportune time to examine the unique role of CHW while there is a global dialogue about how to increase their efficiency and effectiveness.

## Methodology

The general objective of the study was to synthesise qualitative data relating to community-based health workers (sample) about social capital (phenomenon of interest) leading to an improvement in health (phenomenon of interest) that can build on theory (evaluation).

As this was a qualitative meta-synthesis and a large portion of literature about social capital and community health workers is quantitative, the objectives and protocol were formulated using the SPIDER tool [12] and for analysis critical interpretative synthesis [13] was used to guide the data extraction and analysis. This method involved refining the research questions in an iterative approach, as the articles were reviewed, and developing synthetic constructs by repeatedly collapsing the themes or codes to develop smaller sets of categories. The sampling technique was purposeful initially and then theory-based as themes were identified to build on the framework. The synthesising argument was thus developed by critiquing articles to gather evidence of the developed theory until a total of 33 articles remained for the final analysis.

The first stage of the research was a scoping review of primary data, in this case, the books and articles written by the three most commonly named authors in reviews on social capital: Putnam, Coleman and Bourdieu. This provided the keywords required for the search strategy and helped inform the development of the protocol.

The review question was as follows: What is the theoretical basis for the improvements in health seen when community health workers (CHW) serve in the community, attributed to social capital? As literature was reviewed, a secondary question evolved concerning the relationship between the CHW and health system that lead to a separate (unpublished) line of argument. This article is concerned with the primary review question.

### Data collection, extraction and analysis tools

The protocol was registered on PROSPERO, ID = CRD42018084130, but it was not published due to the need to constantly update it as the research process evolved. To manage the large quantity of included papers and for reproducibility, data was coded using qualitative software, QDA Miner Lite version 2.0.3, in two different projects; one examining the health system and CH*W*/*V* which is reported in another article, and the other examining social capital and CH*W/V* which is presented here.

### Inclusion criteria

Research and commentary set in communities without secondary or tertiary level health systems who have community health workers and volunteers (sample) reporting on any aspects of social capital leading to health improvements including, but not limited, to social cohesion, participation, social networks (in the context of face-to-face contacts, not Internet), sense of belonging, increased health knowledge, trust, resilience, social determinants of health, reciprocity and models or theories in relation to groups of unrelated people. Other phenomena of interest were data relating to improvements in health through working in groups or with communities with CHW, examining the dynamics referred to as social capital, social cohesion, participation or empowerment (or reasonable equivalents of these). Although the data sought was qualitative, the reviewers considered all study designs and research types, with no restriction on publication date. Relevance was measured by the presence of qualitative data and the ability to contribute to theory or development of a model. Exclusion criteria were also elaborated extensively and are available on the registered protocol at this link (https://www.crd.york.ac.uk/prospero/display_record.php?RecordID=84130).

### Search strategy

The following keywords were used in combination: *social capital*, *empowerment*, *social cohesion*, *community participation*, *community groups*, *community health worker*, *community health volunteer + model*, *theory*, *health*, *qualitative.* One reviewer conducted a search of PubMed using the keywords with no date limit, yielding a total of 2635 papers. The papers were then screened based on the inclusion and exclusion criteria, and 147 were selected for full-text retrieval. They were then entered into QDA Miner Lite v2.0.3 software and coded with the purpose of finding frequencies of themes in the literature and to understand the concepts from the point of view of the authors. A second search was then widened to find papers relating specifically to CH*W*/*V* and the themes already generated by searching PubMed, Science Direct, Google Scholar and hand searches of reports, documents and theses resulting in a total of 442 full-text articles (including the original 147) that fit the selection criteria.

**Fig. 1:**
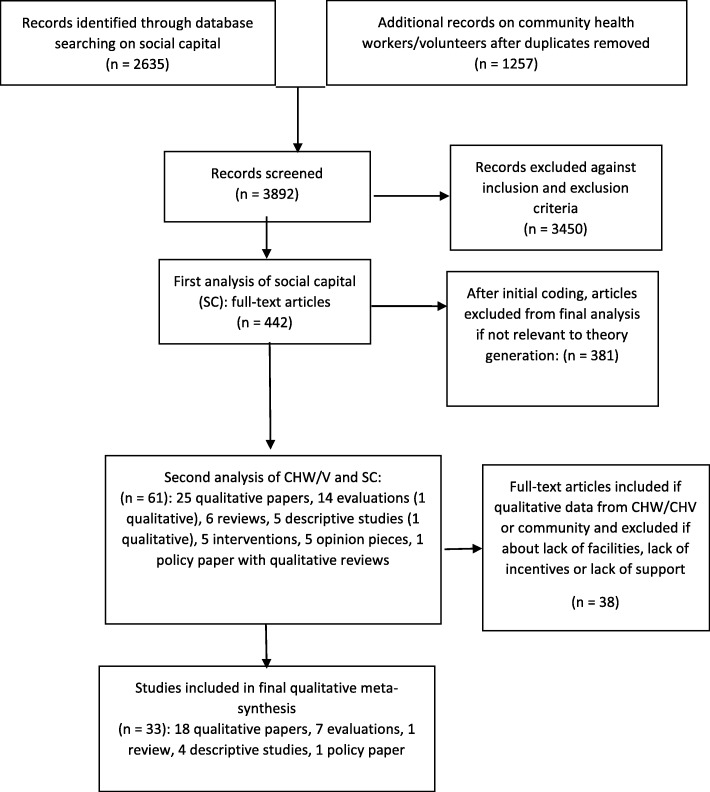
Flow diagram of sourced articles

The 442 articles were also coded into the qualitative software QDA Miner Lite v2.0.3 for their contributions to social capital theory as described below under results. In order to collect evidence for the line of argument that was being developed, these articles were then screened for their relevance to theory generation for community health systems, resulting in a total of 61 articles. These were critically reviewed for assumptions and context with the results of this analysis re-iterating the importance of hearing the perspective of the community health worker or volunteer (CH*W*/*V*) and community. The reviewers further narrowed the inclusion criteria to those papers with direct qualitative data from CH*W/V* or community members for final analysis. After rejecting papers that did not fulfil this criterion, the final number of papers for in-depth critical interpretative synthesis became 3433 (see Fig. 1).

### Quality

The quality of papers selected for social capital theoretical content was very loosely assessed as the reviewers were looking for qualitative data and interpretative discussion; the presence of original qualitative data was usually sufficient for inclusion once other criteria were satisfied. According to Dixon-Wood’s (2006) guidelines, the authors were aware of the need to be more flexible so as not to exclude relevant concepts, even in a paper that may have methodological weaknesses. Usually publication bias is avoided by endeavouring to access unpublished theses, conference papers and other ‘grey’ source materials; however, our initial analysis uncovered the dichotomy between the needs of the community and the expectations of those outside the community, including those who make decisions about what is written and what is published. Therefore, it was decided to include only published papers in the final analysis, although grey source material did inform the themes and framework until this point. Due to the large volume of papers reviewed, only the most pertinent references are quoted below. Full lists of references are available upon request.

## Results

Studies in communities with CH*W*/*V* found that social capital is generated through CH*W/V* using their networks and the development of relationships of trust to overcome obstacles [7]. These networks include traditional healers, friends and families, suppliers of resources (shopkeepers, farmers) and other ‘hidden players’. Poverty’s role in social capital theory is contentious. Although CH*W*/*V* use their social networks and social trust to confront issues related to poverty, there are also unrealistic expectations on the part of the community that they will be able to solve the problems caused by poverty, by supplying food, medicines, transport or money [14]. In order to maintain their trust with the community, CH*W*/*V* will often use their own resources to fulfil these expectations [15].

Using the steps of critical interpretive synthesis [13], the analysis critiqued the articles from the point of view of the already established constructs of relationships, networks and trust between the actors in community settings, resulting in the development of three synthetic constructs that explained the unique health promoting features of community-based health workers: *service as a calling, accompaniment and empowerment*. These were tested using the 33 articles that contained direct qualitative data from CH*W*/*V* or community members.

### Representativeness of the final articles

Of these 33 articles, the dates of publication started from 2005 and the majority (18) were qualitative inquiries involving interviews with CH*W*/*V* or community members. An analysis of the ‘voice’ of the paper to ensure relevance and generalisability, with some papers representing more than one perspective, found that 31 were written from the CH*W*/*V* point of view and 19 were written from the perspective of health services and 19 from the perspective of NGOs or trainers and supervisors of CH*W/V*. Sixteen papers included the perspective of the community or service users, 11 were from a policy perspective, two from a research point of view and only one from economic perspective. The countries of origin of CHW in the articles included Afghanistan, Angola, Bangladesh, Brazil, Ethiopia, Ghana (3), Guatemala, Haiti, India (3), Iran, Kenya (3), Malawi, Nepal, Rwanda, South Africa (4), Swaziland, Tanzania, Uganda (3), the United States of America, multi-country Africa (2) and two global reviews. The author affiliations of the countries of origin were also analysed; however, many of the authors were affiliated with a university in another country but originated from the country of the study, thus this analysis was not found to be useful.

### Service as a calling

When examining motives of the CH*W*/*V*, three of the four most common themes consistent across all papers were the *respect* given to the role of CH*W*/*V*, *religious beliefs or love for the community* and the attitude of *caring, service or social obligation* to care for others. CH*W/V* stated that their service was a calling which they loved [16]. In many cases, being a CH*W/V* was an opportunity to serve the community for people who otherwise had few other employment opportunities [15]. Community members see CHV as vital helpers who act in the patient’s best interest; however, when service becomes work and CHV become CHW, then trust is decreased. The following quotations are from interviews with volunteers, although they are labelled as CHW:

"In some cases, the clients demanded painkillers, or refused to believe the CHWs were volunteers, and insisted that they share their pay with them, levelling accusations that ‘we have eaten the money’ … They complain that our job is just registering them all the time without any pay." [17], p. 10

"The insider identity prevented the CHW status from being labelled as something different, special, or ‘more’. The CHW role was framed around that of ‘mere’ volunteer work carried out in service of community … In this regard, it could be seen as embodying the spirit of ‘ubuntu’, or humanness and kindness, …. The work of CHWs was also motivated by the fact that they came from impoverished communities with little access to employment and other work opportunities." [15], p. 1660

"CHWs described their work as a ‘calling’ and expressed their love for volunteering and public service with statements such as:‘I love being a person who serves others’ (CHW16, age 50, male) and, ‘I love my job’ (CHW1, age 35, male)." [16]. p.59

### Accompaniment

Community health workers and volunteers see themselves as agents of change who accompany the members of the community as they change their lives [18]. They use their local knowledge to explain health in simple terms and can address traditional false beliefs [14, 16]. However, they acknowledge that information alone is not enough to change behaviour; it is their care and concern for the community that motivates behaviour change. Regarding traditional beliefs, the models and theories of social capital acknowledge the negative effects of social capital when a network of trusted members reinforces negative or unhealthy social norms [19]. The role of the CH*W*/*V* as part of a trusted network is to address and change these social norms from within using empathy, persistence and patience [20].

""All these changes that you see in [the] rural environment are the result of health education provided by us. People’s beliefs and behaviour changed a lot and it makes everything easier for us." Male *CHW* [19]. p. 2289

The community health system’s strength is the ability to use ‘hidden’ networks, not known or recognised by ‘outsiders’ and to adapt to local conditions. CH*W*/*V* also accompany other actors in the community, including traditional healers and other members of health teams, working well with heterogenous groups and in diverse settings [21, 22].

"CHWs reported that many patients demonstrate unwavering faith in traditional healing and religion, in which case discouraging the use of herbal remedies or prayer could conceivably erode patients’ trust in the healthcare system … To better reach this patient population and reduce competition-related barriers to patients’ linkage to and retention in ART, CHWs suggested that the healthcare system join forces with traditional healers and religious leaders through education and collaboration. …CHWs also felt that education campaigns could help patients living in communities with strong traditional influence begin to recognize their HIV-related symptoms as having a biomedical, as opposed to spiritual, origin." [23], p. 388

The mistrust of ‘outside’ health services and NGOs can be understood using the same construct of accompaniment or being with the community. A sudden lack of funding, a change in priorities or inability to keep promises is seen as ‘them’ no longer being with/accompanying ‘us’. The trust lost from such behaviours cannot be regained easily [24].

A large part of the CH*W*/*V* role is beyond their scope of practice as defined by the training organisation and is consistent with the concept of accompaniment. Papers reviewed from each continent described how CH*W*/*V* would cook, clean, bathe, feed and give medications to community members [25].

Trust remains a central issue in regards to accompaniment; the sense that the community trusted that the CH*W/V* would be with them if they took an action. For example, an evaluation of the uptake of a malaria programme found that there were no differences in the knowledge, perceptions or understanding of the chemoprevention offered between those who took up the programme and those who had suboptimal uptake. The facilitators of uptake were rather the trust that the caregivers had for the medical experts and CHWs, the community networks that reinforced the usefulness of the programme and the learnt trust of those who were in the programme [18]. Trust is determined by the values of cooperation, empathy and open communication and was the basis of defining (in the eyes of the community) a good or bad health worker [26].

The construct of accompaniment is not simply ‘being one’ with the community. The CH*W*/*V* and the community members both stressed that their role is respected and separate to being a normal member of the community. This enables them to accompany community members who lack autonomy and decision making power by working with husbands and religious leaders [27].

"…when they say that their men [Husbands] do not allow them to use contraceptives…next time I go to their house at a time that her man is at home, and I ask her to call her man too, and counsel them together." Female CHW [28], p. 7

### Empowerment

The other most common motivation for becoming a CH*W*/*V* was perceived incentives in the form of financial, educational or other material goods that would help the CH*W/V* improve the condition of their family and community. On further analysis, this stemmed from the perceived lack of control over the social, economic and physical condition of the community which could only be transformed through outside help (knowledge, money or resources). The relationship between the community and outside authorities is referred to as linking social capital and formed another element of this study reported elsewhere.

The CH*W*/*V* felt that their role enabled their families and communities (and themselves) to become empowered.

"Although volunteers complained that they lacked monetary support to undertake their activities and health workers also agreed with this view, all the volunteers were committed to continuing volunteering. This was mainly because the volunteers perceived that they were benefitting from the work as a volunteer and saw themselves as empowered women." [29], p. 7

Empowerment is a goal or vision for most CH*W*/*V* who are constrained by the social norms of the community. These constraints are seen more acutely when CH*W*/*V* are from different social, religious or geographical backgrounds than the people they serve [27]. CH*W/V* of both genders are also expected to maintain their roles as spouses, parents and workers (if they are already working on their farms, for example). Some female CH*W/V* manage with the support of their families, but many find they can only serve part-time [30].

"… my husband rebukes me as: What is the point of working for community, when you can’t even take care of your own family?" [31], p. 8

"A few CHWs also shared that the programme gave them a higher status despite their gender. Some claimed that having this position had given them more authority in the household and more autonomy over household purchases. Many of the …(CHW)* who were trained on family planning had started using family planning methods themselves, and served as role models for the women in the community." [31], p. 4 (*authors’ addition)

In other countries, CHW are predominantly men due to high unemployment and lack of other job opportunities [32]. In this situation, empowerment is experienced in the ability to be a guide for the community and improve the financial condition of their own families.

Although the term CHW is often used for CHV in the literature, the issue of economic empowerment is where the largest differences are seen between those who are paid regularly and volunteers. For example, in India, ASHA (CHV) claim that Anganwadi workers (CHW) receive regular income independently of how much work they do, whereas they receive incentives only for specific tasks, such as accompanying women to hospital for deliveries, and are expected to perform other, more time-consuming tasks of health education and home support, without any incentives [33]. In Ghana, CHV provide preventive and curative services for which they charge the community a fee for medicines in lieu of a salary; they are not linked to the formal health system so that the community does not get improved access to formal health care and they have to divide their time between volunteering and income generation as they live in a condition of poverty [34].

Apart from the gender and financial aspects of empowerment, from the point of view of the community and CHW living within the community, other social determinants of health need to be addressed including access to transport, the availability of trained staff at health services [35] and basic facilities such as water and electricity [32, 34]. Developing social capital and improved health were not enough, the community and CH*W*/*V* expect ‘outside authorities’ to also address economic conditions, infrastructure and inequity [36].

## Discussion

This review was conducted to add to theory development about cause of the effectiveness of community-based health workers and volunteers (CH*W*/*V*) in changing health behaviours, using social capital as a framework. The tool used, critical interpretive synthesis, aims to question the assumptions in the literature and examine the context [37]. This review found a dichotomy of needs and expectations between the policies, programmes and agendas of CHW programmes and the CH*W*/*V* and community. What is significant is that even when the expressed needs and expectations of CH*W/V* for training, supervision and resources are not met, they still manage to improve health behaviours of the community due to their unique motivations, attitudes and behaviours conceptualised in the three constructs of service, accompaniment and empowerment.

The first clear construct was seeing their service to the community as a calling. Some theories of social capital reduce human behaviour and relationships to equations of benefit, risk and expectation [38]. Our findings are that in a community health system, qualities or virtues that are just as elusive when it comes to defining and measuring them as social capital, are the strongest motivators of CH*W*/*V*, beyond financial benefit or respect and stature in the community. These qualities are either inbuilt, for example the CH*W*/*V* who believe they must give back to the community for altruistic or religious reasons; or are created by the love and respect they receive for their services.

The next construct was accompaniment, which differs from the service delivery model of healthcare. Some of the unique features of the care given by CH*W/V* that illustrate this are home-based care, improve adherence due to their caring relationship with families, knowledge of family members or friends, use of informal support systems and being an example of good health behaviours. On the other hand, absence of any of these attributes decreases trust and adherence, particularly when CHW are unable to deliver services, are perceived as getting benefits that they are not passing on the community or do not live up to expectations. In this sense, an unpaid volunteer can create more social capital through trust as they are thought to be motivated by service, whereas a paid CHW who has a high workload and is under-resourced faces barriers in developing trust in the community.

The third construct was the notion that empowerment is the goal for the individual CH*W*/*V*, their families and communities, embracing all the other inequalities that communities face from transport, electricity and water shortages to the need for accessible, high-quality education, employment and health care. Farquhar et al. state that increasing social capital in communities through building trust and networking should not be seen as an alternative to bringing about equity in the social determinants of health [36].

The empowerment construct expressed by the voices in the reviewed articles is inclusive for the whole community: the CH*W*/*V* also want to involve traditional and social leaders in the process, acknowledging their role in social and cultural norms, and desiring that they also become aware and educated about health behaviours. Empowerment of the CHW and CHV is potentially an outcome of their role through education and improved financial status, but can also be thwarted if their workload or lack of incentives creates more pressure on their families.

From the results, we postulate that the CH*W*/*V* cannot be separated from the community in which he or she serves, the way a doctor or nurse, who have very specific service delivery skills, can be placed into any environment. Communities want curative services and CHW have the ability, with adequate training, supervision, resources and links to health services, to provide that. Communities also need social determinants of health, issues of equity and social welfare to be addressed if they are to be empowered to be protagonists of their own health. We propose that these are two very different but complementary skill sets. CHV who do not have adequate training or supervision can be a risk to the community and make them less effective [39]: a study in Ghana found that while child mortality improved with community health nurses, it slightly worsened in areas with only CHV [40].

On the other hand, there are also the social justice issues involved in a workforce that is not paid and has unclear expectations from both the community and agencies that train them. Studies have shown that the use of incentives versus regular payments leads to a breakdown of trust on all sides [41]. Unlike some authors [42], we do not believe that the answer is to stop unpaid volunteerism, but we do acknowledge the financial and social strain that the lack of incentives brings. By limiting the role of CHV to non-clinical primary health care and limiting the time burden required from CHV, a larger number of community members can be trained and serve the community in health promotion roles, while developing a larger pool from which to select paid CHW.

This model has been used successfully in Indigenous health contexts where CHV are cultural mentors [43] and in Rwanda [44], where there are teams of two CHW for general health-related tasks and one CHW who is responsible for social and welfare tasks. In other countries with both CHW and CHV, their roles are different, but not as clearly defined.

The implications for programming would be a cadre of workforce (CHW) for whom investment in training, supervision and provision of resources is essential to enable efficient delivery of service and maintain trust with the community. The training of CHW would have to equip them with skills in accompaniment and empowerment, as identified in this review. The gap in our learning for which further research is required is how to engage, train and supervise the volunteer cadre of health workers at the community level. In line with the findings of this review, service, accompaniment and empowerment related skills, such as advocacy, counselling and working with groups could be possible areas for training.

## Conclusion

The literature makes it clear that there is a need for trained community health workers to deliver diagnostic and curative services to communities, and the results of this review highlight the social capital built through the trust that exists between CHW and the community when they are able to fulfil this need through training and provision of supplies. This is consistent with the model of social capital in which relationships of trust are built through expectation and reciprocation or obligation.

The results also indicate that throughout the world there are people in communities who are willing to volunteer their time to serve and empower their communities as long as they have the time to also earn an income and support their families. In many cases, this is through agriculture or other paid work. These part time volunteers also generate social capital by performing social action for the common good. In both cases, health behaviours are changed as a product of the social capital produced.

Recent reviews of CHW have focussed on their supervision, incentives and integration into the health services [45]. The other unique features of the community-based health worker role described above are essential to preserve in programme development so that CHW can continue to foster changes in behaviour that promote health in vulnerable communities; however, the question remains as to how to plan for these constructs in CHW programmes. Service, accompaniment and empowerment require time rather than financial or intellectual resources. One of the common complaints of CH*W*/*V* is that they feel overburdened and under supported. One solution proposed is to streamline the work of CHW and CHV so that they both have time for these essential elements of their community health roles. This concept is new and requires further field research and dialogue based on the outcomes of behaviour change and community empowerment, rather than narrow health-related indices.

## Abbreviations

ART: Anti-retroviral treatment
CHV: Community health volunteer
CHW: Community health worker
CH*W*/*V*: Community health workers and volunteers (paid and voluntary)
HIV: Human immunodeficiency virus
NGO: Non-governmental organisation
SPIDER: Sample, phenomena of interest, design, evaluation and research type (qualitative tool for study designs)
WHO: World Health Organization
